# A fast optimization approach for treatment planning of volumetric modulated arc therapy

**DOI:** 10.1186/s13014-018-1050-x

**Published:** 2018-05-30

**Authors:** Hui Yan, Jian-Rong Dai, Ye-Xiong Li

**Affiliations:** Department of Radiation Oncology, National Cancer Center/Cancer Hospital, Chinese Academy of Medical Sciences, Peking Union Medical College, Beijing, 100021 China

**Keywords:** Volumetric modulated arc therapy, Fluence-map optimization, Leaf-sequencing

## Abstract

**Background:**

Volumetric modulated arc therapy (VMAT) is widely used in clinical practice. It not only significantly reduces treatment time, but also produces high-quality treatment plans. Current optimization approaches heavily rely on stochastic algorithms which are time-consuming and less repeatable. In this study, a novel approach is proposed to provide a high-efficient optimization algorithm for VMAT treatment planning.

**Methods:**

A progressive sampling strategy is employed for beam arrangement of VMAT planning. The initial beams with equal-space are added to the plan in a coarse sampling resolution. Fluence-map optimization and leaf-sequencing are performed for these beams. Then, the coefficients of fluence-maps optimization algorithm are adjusted according to the known fluence maps of these beams. In the next round the sampling resolution is doubled and more beams are added. This process continues until the total number of beams arrived. The performance of VMAT optimization algorithm was evaluated using three clinical cases and compared to those of a commercial planning system.

**Results:**

The dosimetric quality of VMAT plans is equal to or better than the corresponding IMRT plans for three clinical cases. The maximum dose to critical organs is reduced considerably for VMAT plans comparing to those of IMRT plans, especially in the head and neck case. The total number of segments and monitor units are reduced for VMAT plans. For three clinical cases, VMAT optimization takes < 5 min accomplished using proposed approach and is 3–4 times less than that of the commercial system.

**Conclusions:**

The proposed VMAT optimization algorithm is able to produce high-quality VMAT plans efficiently and consistently. It presents a new way to accelerate current optimization process of VMAT planning.

## Background

VMAT is widely used in cancer treatment in radiation oncology departments due to its high efficiency in treatment delivery [1]. Compared to conventional IMRT, VMAT delivers radiation dose while MLC leaf, dose rate and gantry move simultaneously [2]. It uses less treatment time and total monitor units (MU) compared to conventional IMRT technique [3]. The predecessor of modern VMAT is intensity modulated arc therapy (IMAT) which was first developed by Cedric Yu in 1995 [4, 5]. It was motivated from the idea of delivering plans with a large number of gantry positions. The fluence map of the beam is pre-calculated and decomposed to several apertures. These apertures is then delivered at a given gantry position by multiple arcs. IMAT requires more arcs which causes extended treatment time. Direct aperture optimization (DAO) was thus proposed to handle the complexity of VMAT optimization using stochastic approach. The stochastic approach is computationally intensive and time-consuming [6–8]. Later, Otto presented an iterative algorithm for VMAT optimization [9, 10]. This algorithm employs progressive sampling strategy and aperture-based algorithm for VMAT optimization where high-quality dose plan can be achieved by a single arc. This technique is successfully adopted in commercial treatment planning system and has been applied to a wide range of treatment sites [11].

Compared to IMRT, VMAT presents a complex optimization problem because of the significantly increased number of plan parameters such as gantry angle, MLC leaf position, dose rate, etc. [12, 13]. Various approaches were proposed attempting to solve this problem. Most of them are evolved from approaches originally developed for IMRT [14–22]. Currently, most of commercial treatment planning systems provide VMAT optimization function and heavily rely on DAO algorithm. RapidArc (Varian Medical System, Palo Alto, CA, USA) employs a progressive sampling strategy and simulated annealing-based DAO algorithm for VMAT planning [9]. Due to the nature of stochastic approach, the optimization process is time-consuming and the optimization result is seldom repeatable. As reported the maximal DVH variation could be up to 2% for OARs using RapidArc [10]. SmartArc (Philips Healthcare, Inc., Thornton, CO, USA) utilizes an IMRT plan (consisting of equal-space fields) to initialize the arc plan. The fluence maps of IMRT plan are then segmented and redistributed to their neighboring angles. A local gradient-based optimization approach is used to fine-tune these apertures to meet the mechanical constraints for actual delivery [16, 17]. In this approach, The fluence maps of all beams are actually derived from few static beams and not fully determined by fluence map optimization (FMO) algorithm.

The purpose of this study is to develop a high-efficient optimization approach for VMAT planning. The remainder of this paper is organized as follow. The progressive sampling strategy is first introduced and the optimization process is described. Then, two important components, fluence-map optimization algorithm and leaf-sequencing algorithm, are explained in brief. These algorithms were developed on an in-house developed treatment planning system. Three typical clinical cases (head and neck, lung, prostate) were evaluated on both in-house developed and commercial treatment planning systems. The dosimetric quality, delivery efficiency, and running time of VMAT plans were then assessed. Finally, the advantage and disadvantage of this approach are discussed.

## Methods

### Progressive sampling strategy

**Fig. 1:**
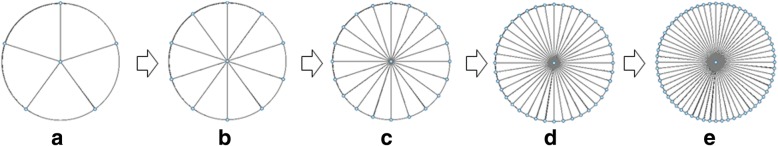
The illustration of progressive sampling scheme. **a** The first beam set with 5 control points (beam spacing 72°). **b** The second beam set with additional 5 control points (beam spacing 36°). **c** The third beam set with additional 10 control points (beam spacing 18°). **d** The fourth beam set with additional 20 control points (beam spacing 9°). **e** The fifth beam set with additional 20 control points (beam spacing 6°)

Most of VMAT optimization algorithms model Linac source as a series of static gantry positions. The MLC positions and MU setting is then determined at each gantry position. Provided the complexity of VMAT optimization, to limit the scale of problem a progressive sampling strategy is employed in this study. It starts with a coarse sampling of gantry positions, then move to fine sampling of gantry positions. An example is demonstrated as shown in Fig. 1. At the beginning of VMAT optimization a coarse sampling of the gantry positions is used to model the gantry rotation range. The initial 5 beams with equal-space are chosen for the first optimization stage. The MLC positions and MUs for the initial 5 beams are achieved by the fluence-map optimization and leaf-sequencing algorithms. Next, the sampling resolution is doubled. And another 5 beams are added to the plan and optimized. The sampling resolution is continuously increased and more beams are added to the plan until the total number of beams is arrived. As shown in Fig. 1, the number of new beams in 5 optimization stages is 5, 10, 20, 40, and 60, while the corresponding beam spacing is 72°, 36°, 18°, 9°, and 6°.

**Fig. 2:**
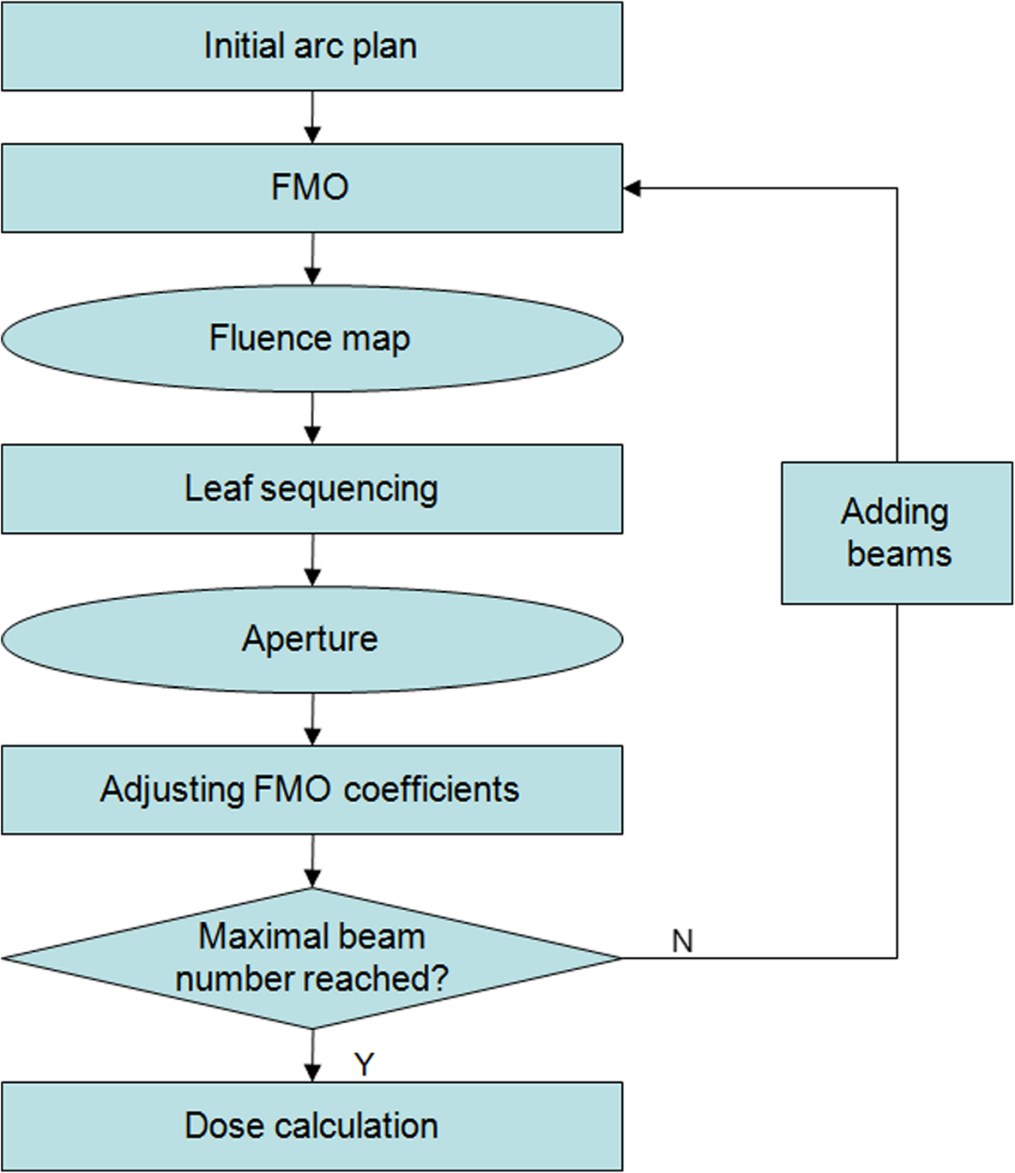
The flowchart of the proposed optimization algorithm for VMAT treatment planning

A VMAT optimization approach was developed based on the existing fluence-map optimization and leaf-sequencing algorithms provided by an in-house developed treatment planning system [23]. The flowchart of VMAT planning proposed in this study is presented in Fig. 2. First, the basic plan parameters such as couch angle and arc range are set by planner. An initial plan consisting of few beams with equal-space is created and their fluence maps are generated by FMO algorithm. These resulting fluence maps are then processed by the leaf sequencing algorithm, and the optimal MLC positions and MUs are determined. Next, the coefficients of FMO algorithm are adjusted based on plan objective and known fluence maps of these beams. In the next round, the sampling resolution is increased and more beams are added to the plan. The optimization process continues until the maximal number of beams is arrived.

### Fluence-map optimization

A gradient-based optimization approach, fast monotonic descent (FMD) algorithm is employed for VMAT optimization. Due to the nature of gradient-based algorithm, the global minimum of objective function can be found quickly. The goal is to find minimum of objective function subject to non-negative fluences for all pencil beams. A non-synchronous updating scheme is used which allows only one pencil beam adjusted at a time. The detail of classic FMD algorithm is described in references [23–25]. To accommodate the progressive sampling strategy, FMD algorithm was modified to account for the varied number of beams in multiple optimization stages. The formulation of the modified FMD algorithm is described in Appendix 1. In brief, new beams are added to the plan in each optimization stage and their fluence maps are optimized with the fluence maps of old beams known. Here, *x*^*new*^ denotes the fluence map of new beam which is unsolved in current stage and *x*^*old*^ denotes the fluence map of old beam which is solved in previous stage. For a given voxel *v*_*ijk*_, its dose is calculated by the sum of dose contributed from *x*^*new*^ and *x*^*old*^. For solving *x*^*new*^ the coefficients of FMD algorithm is adjusted based the planning dose of target and the dose contribution from *x*^*old*^. As fluence map of *x*^*new*^ known, it is processed by a leaf sequencing algorithm and a uniform fluence map for a single aperture is obtained. The uniform fluence map replaces the original fluence map of *x*^*new*^ and is used as the final value of *x*^*new*^.

### Leaf sequencing

Several leaf sequencing algorithms were developed in the past years such as powers of 2, linear programming, and graphics searching [26–29]. These algorithms decompose fluence map into several apertures with uniform fluences. Given the dynamic feature of beams in VMAT, the leaf sequencing algorithm has to deal with more mechanical constraints related to gantry speed, leaf speed, and dose rate than those of static beams. Mixed integer linear programming (MILP) was successfully applied in many industrial applications due to its capability in handling large-scale optimization problems [30, 31]. In this study, a MILP model was developed using a commercial optimization software package (IBM ILOG CPLEX). It assumes that the fluence map of a beam at a given control point is a uniform fluence map for a single aperture. The aperture of the new beam is determined subjected to the apertures of the two old beams closet to it (one is at front side and one is at end side). As the original flence map of *x*^*new*^ is non-uniform, it is necessary to convert it to a uniform fluence by a leaf-sequencing algorithm with certain constraints. These constraints are mostly related to mechanical limitation of Linac and MLC and described in Appendix 2. Due to the nature of progressive sampling strategy, the beams solved in the earlier stages have larger apertures and weights than the beams solved in the latter stages. This is because the number of beams is less in the earlier stages and more in the latter stages. The MILP model is described in Appendix 2. The goal of this model is to search for an optimal solution, uniform fluence map, from a non-uniform fluence map under certain constraints. The MILP problem is solved using branch-and-bound technique provided by CPLEX software. As an example of fluence map consisting of 30×30 bixels, the total number of constraints is ~ 1800 and the total number of independent variable is ~ 2000. The processing time is approximately 0.5 s per beam.

### Plan evaluation

Three clinical cases were selected for evaluation including head and neck, prostate, and lung. The patient CT images and contours were exported from Pinnacle (Philips Healthcare, Inc., Thornton, CO, USA). Then they are segmented into regions of interest (ROIs) for plan optimization using an in-house developed treatment planning system. In this study, the simulated leaf width is 0.5 cm and the leaf step is 0.5 cm, which resulted in beamlet size of 0.5×0.5 cm. The range of leaf speed is 0.5–1 cm per degree because the leaf speed is 3–6 cm per second and gantry speed is 6 degree per second. The prescription dose for PTV and dose constraints for OARs are the same for both IMRT and VMAT plans for each case. A single dynamic arc plan consisting of 180 beams and 2° spacing was created and fluence maps were optimized by the proposed VMAT optimization algorithm on the in-house developed system. The numbers of beams in five optimization stages are 15, 30, 60, 120, and 180, while the corresponding beam spacing is 24°, 12°, 6°, 3°, and 2°. A corresponding IMRT plan consisting of 9 equal-space fields was created and fluence maps were optimized by FMD algorithm on the in-house developed system. The dosimetric quality of plans is quantified by conformity index (CI), homogeneity index (HI), quality score (QS) [32], and weighted root mean-square error [23], which are defined as below.

CI = V_T*V*_/V_95%_, where V_TV_ is the target volume and V_95%_ is the volume corresponding to 95% dose prescription. If V_95%_ = V_TV_, CI =1 which is perfect.

HI = D_5%_/D_95%_, where D_5%_ and D_95%_ are doses received at least 5 and 95% target volume respectively. if D_5%_ = D_95%_, HI =1 which is perfect.

Quality score (QS) quantitatively measures the difference between the dose plan and the dose constraint of all anatomical structures. $\mathrm{QS}\kern0.5em =\kern0.5em \sum \limits_j\left|\frac{\left({M}_j-{C}_j\right)}{C_j}\right|$ if objective is violated by plan dose, where *C*_*j*_ is the *j-*th dose-volume constraint, *M*_*j*_ is the corresponding plan dose. If no objective is violated, QS = 0 which is perfect.

Weighted root mean-square error (WE) measures the difference between the plan dose and prescribed dose of all voxels and represents the value of objective function. $\mathrm{WE}\kern0.5em =\kern0.5em {\left(\frac{\sum \limits_{i,j,k\in {V}_{TV}}{w}_{ijk}{\left({P}_{ijk}-{D}_{ijk}\right)}^2\kern0.5em +\kern0.5em \sum \limits_{i,j,k\in {V}_{CO}}{w}_{ijk}{\left({P}_{ijk}-{D}_{ijk}\right)}^2+\sum \limits_{i,j,k\in {V}_{NT}}{w}_{ijk}{\left({P}_{ijk}-{D}_{ijk}\right)}^2}{N}\right)}^{\frac{1}{2}}$, where *P*_*ijk*_, *D*_*ijk*_, and *W*_*ijk*_ are defined in Eq. 1, and *N* is the total number of voxels belonging to TV, CO and NT. For a plan which *P*_*ijk*_ = *D*_*ijk*_ for all voxels, WE = 0 which is perfect.

The dose distribution of plans is evaluated based on cross-sectional dose distribution and dose-volume histogram. The total number of segments and monitor unit for both plans are recorded. Additionally, estimated arc delivery times and the computation time of plan optimization are recorded. For demonstration purpose, few important ROIs are used in plan optimization. For head and neck case, they are PTV, spinal cord, left and right parotids and mandible. For prostate case, they are PTV, left and right femur heads, bladder and rectum. For lung case, they are PTV, left and right lungs, spinal cord and heart. All tests are performed on a DELL Optiplex N9010 computer, equipped with Intel(R) i7–3770 CPU and 72GB RAM.

Corresponding to the VMAT plans made on in-house developed system, three VMAT plans made on Pinnacle planning system were assessed. They were made by experienced planners and approved for clinical treatment. These plans are VMAT plans consisting of 2 full arcs with 4° spacing and 6 MV beams computed with convolution superposition algorithm. The dosimetric and delivery statistics of these clinically approved plans are compared to those of IMRT and VMAT plans made on the in-house developed system. Note that the optimization algorithm, the dose calculation engine, the dose-volume constraints and many others of clinically approved plans are different from those of plans made on the in-house developed system. The metric, WS, is not calculated for the clinically approved VMAT plans because the weight specification in Pinnacle system is different. For distinguishing clinically approved VMAT plans from the plans made on the in-house developed system, VMAT plans made on pinnacle system are represented by VMAT_P.

## Results

**Fig. 3:**
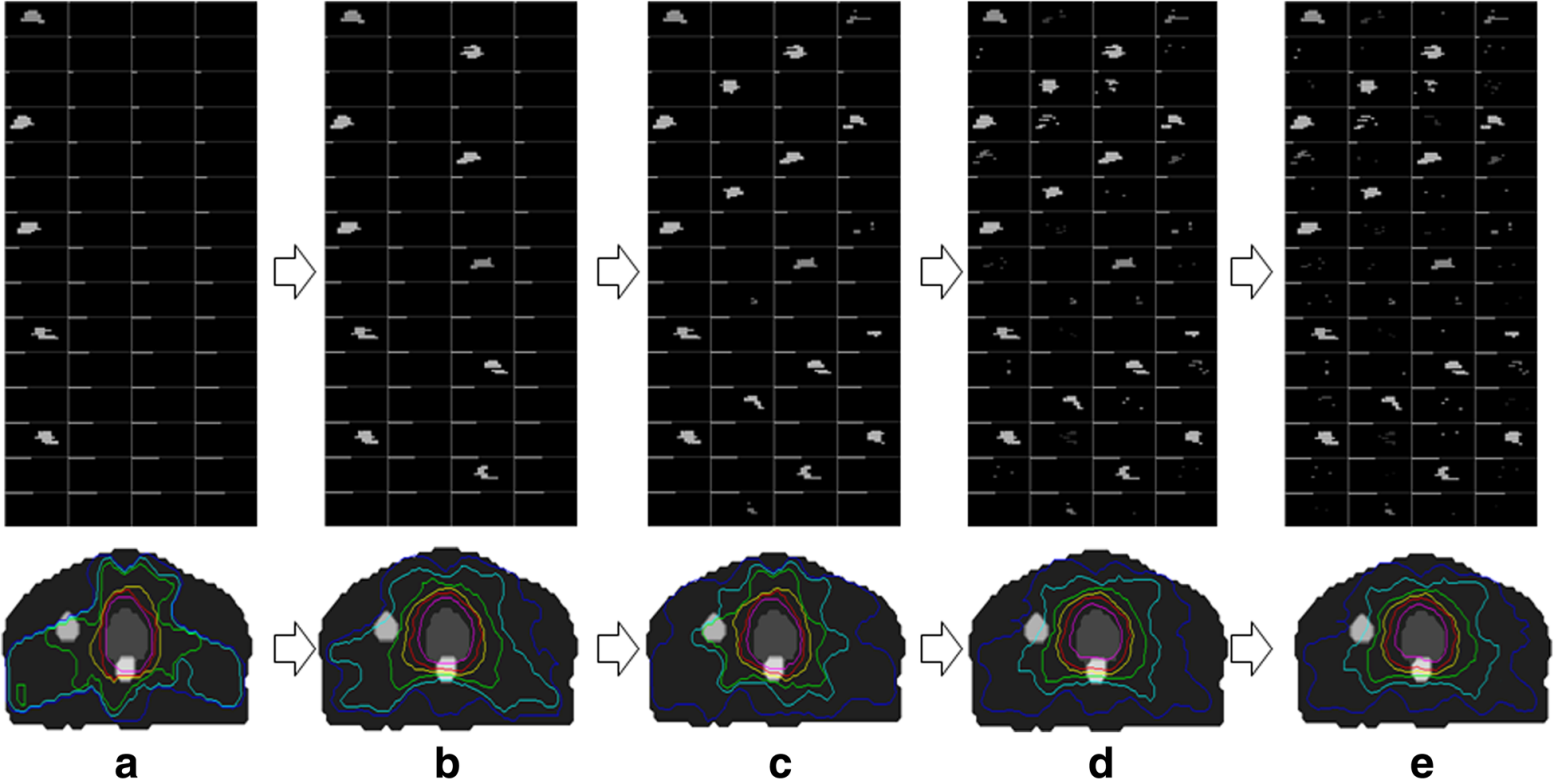
The segments and dose distributions of VMAT plans for five beam sets with the increasing numbers of beams. **a** VMAT plan with 5 equal-space beams (72° spacing). **b** VMAT plan with 10 equal-space beams (36° spacing). **c** VMAT plan with 20 equal-space beams (36° spacing). **d** VMAT plan with 40 equal-space beams (9° spacing). **e** VMAT plan with 60 equal-space beams (6° spacing)

The fluence map and plan dose distribution achieved by progressive sampling strategy is demonstrated in Fig. 3. For each plan, the subplots at the top are the segments of all beams while the subplots at the bottom are the dose distribution in axial view. The grey level of segment indicates the magnitude of its weight. White represents the highest value (255) and black represents the lowest value (0). The corresponding beam arrangements are shown in Fig. 1. In the earlier stages, the values of fluence maps of new beams are larger as there are fewer beams. As more beams included, the values of fluence map of new beams are smaller. The plan dose quality improves quickly in the earlier stages (as shown in Fig. 3b and c), and gradually saturates in latter stages (as shown in Fig. 3d and e.

**Fig. 4:**
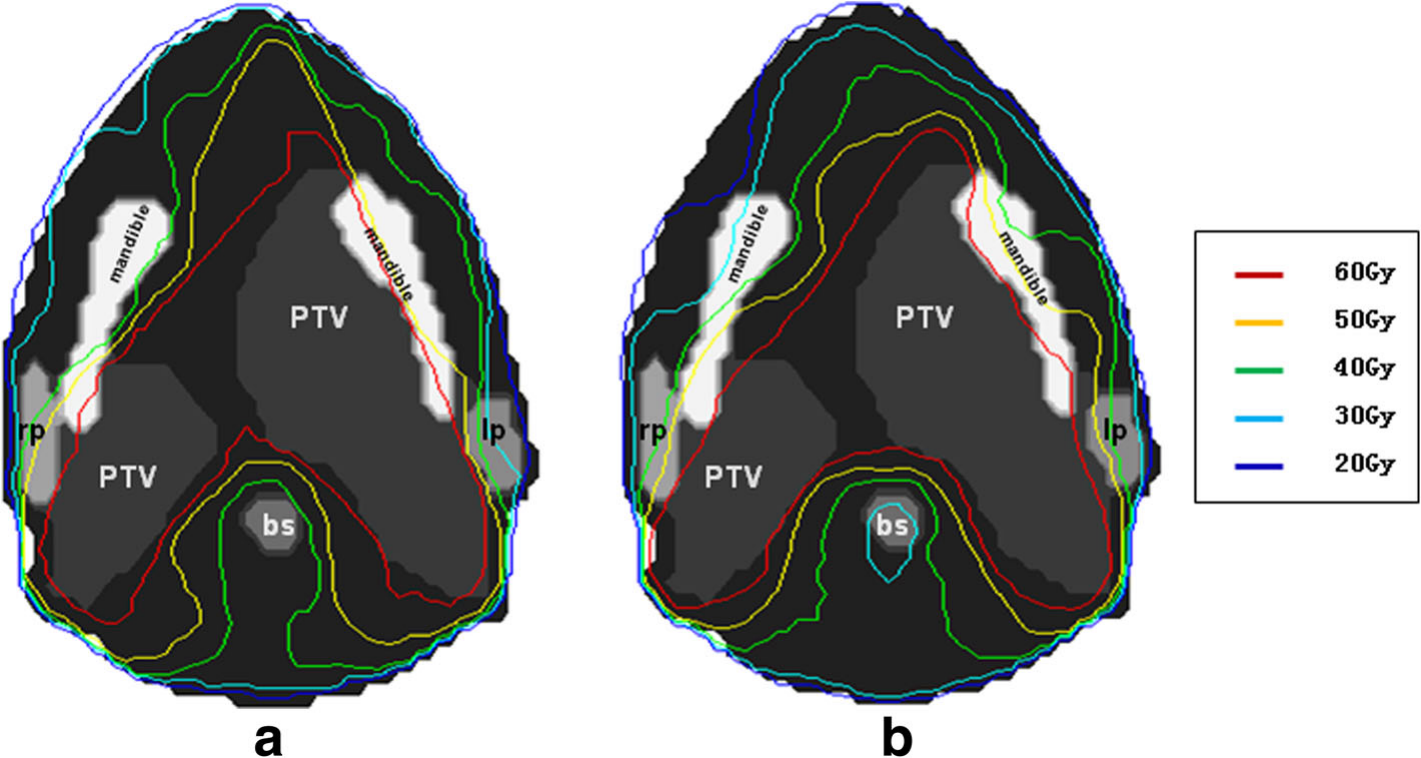
The transaxial plan dose distribution of head-and-neck case in (**a**) IMRT plan and (**b**) VMAT plan

**Fig. 5:**
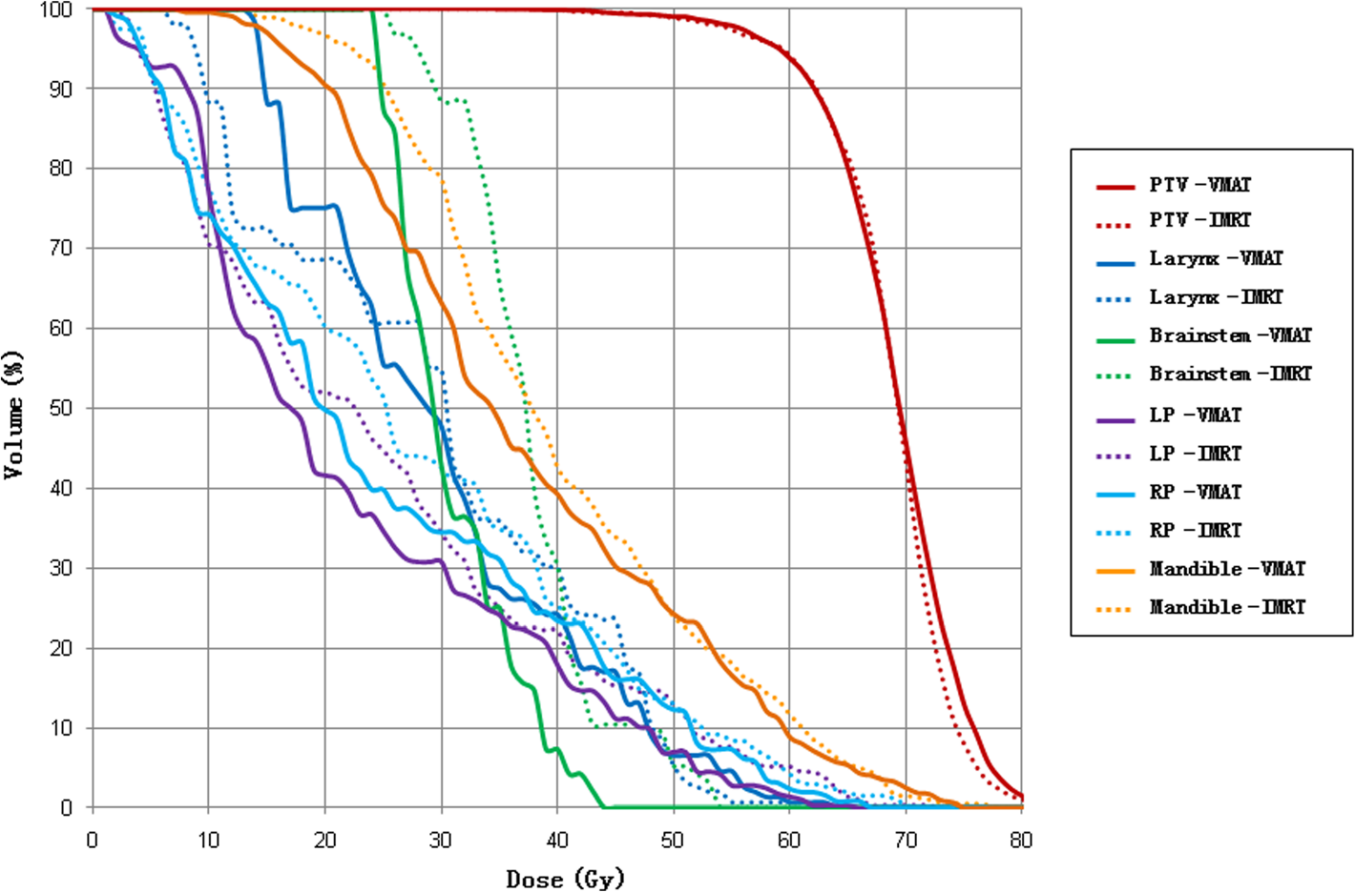
The DVH of head-and-neck case in IMRT plan and VMAT plan

For the head and neck case, the objective is to maintain uniform dose (60 Gy) to the PTV, while minimize the dose to the parotid gland (V30Gy < 50%) and maximize the sparing of the brainstem (Dmax< 54 Gy), the larynx (Dmax<40Gy), and the mandible (Dmax< 60 Gy) as much as possible. Dose distributions for IMRT and VMAT plans are shown in Fig. 4. DVHs for IMRT and VMAT plans are compared as shown in Fig. 5. The VMAT plan is better than the corresponding IMRT plan. The coverage of PTV is similar for both VMAT and IMRT plans. The OAR sparing from VMAT plan is better for brainstem and mandible, but is similar for larynx and parotids (left and right). The max dose to brainstem is reduced by 10 Gy. Dose distribution of VMAT plan is more uniform with fewer hot and cold spots. The QS, WE, CI, and HI for both plans are shown in Table 1. The WE is reduced by 10% for VMAT plan, while QS, CI and HI are similar for both plans. The total number of segments and monitor units for both plans are shown in Table 1. The number of segment is similar for both plans. The total MU for VMAT plan is reduced by 40%. The computation time is 40 s for IMRT plan and 312 s for VMAT plan as shown in Table 1. The estimated delivery time is between 96 and 192 s based on a Varian Trilogy machine with variable dose rate (300–600 MUs per minute). VMAT_P plan show better plan quality and fewer MUs than VMAT plan, but the number of segments and optimization time are higher.

**Table 1:** Comparison of performance between VMAT plan and IMRT plan

Treatment site	Plan type	Plan performance						
		QS	WE	CI	HI	Number of segments	MU	Time (s)
HN	IMRT	0.92	79.91	1.83	1.26	129	1775	40
	VMAT	0.84	71.63	1.82	1.23	108	960	312
Prostate	VMAT_P	0.13	N/A	1.54	1.02	364	810	1080
	IMRT	0.32	63.44	1.43	1.23	112	1418	36
	VMAT	0.31	55.43	1.41	1.22	116	1180	294
	VMAT_P	0.13	N/A	1.21	1.03	182	1076	1210
	IMRT	0.13	15.9	1.83	1.14	166	1178	32
Lung	VMAT	0.13	15.8	1.84	1.12	138	1162	216
	VMAT_P	0.11	N/A	1.63	1.03	108	626	760

**Fig. 6:**
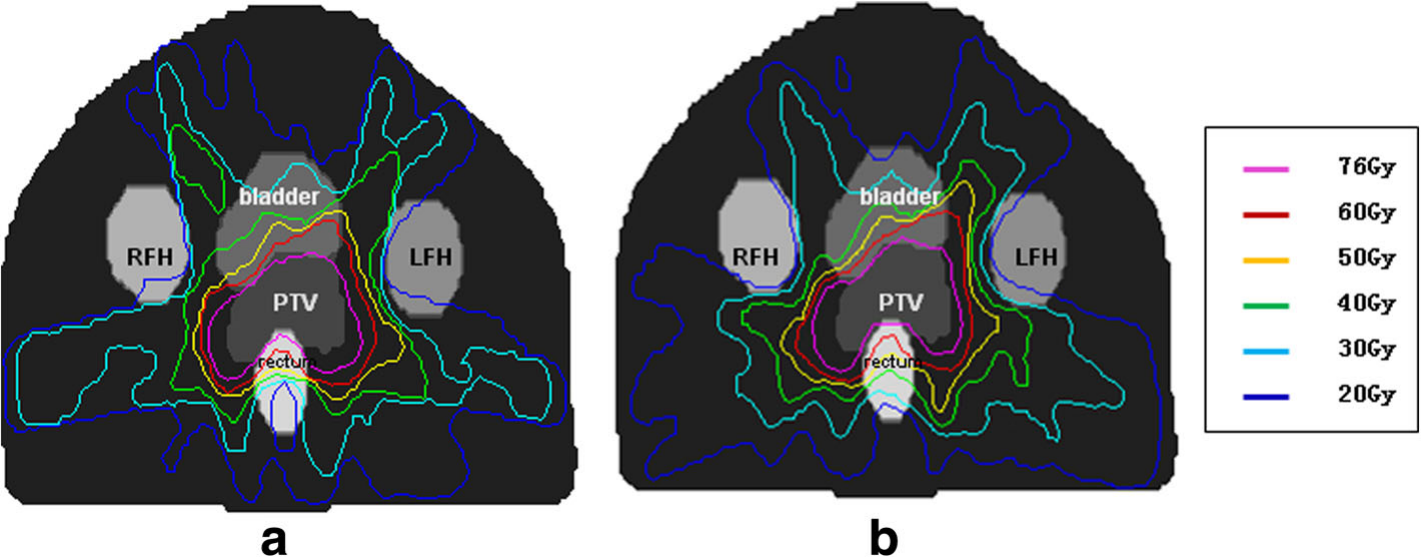
The transaxial plan dose distribution of prostate case in (**a**) IMRT plan and (**b**) VMAT plan

**Fig. 7:**
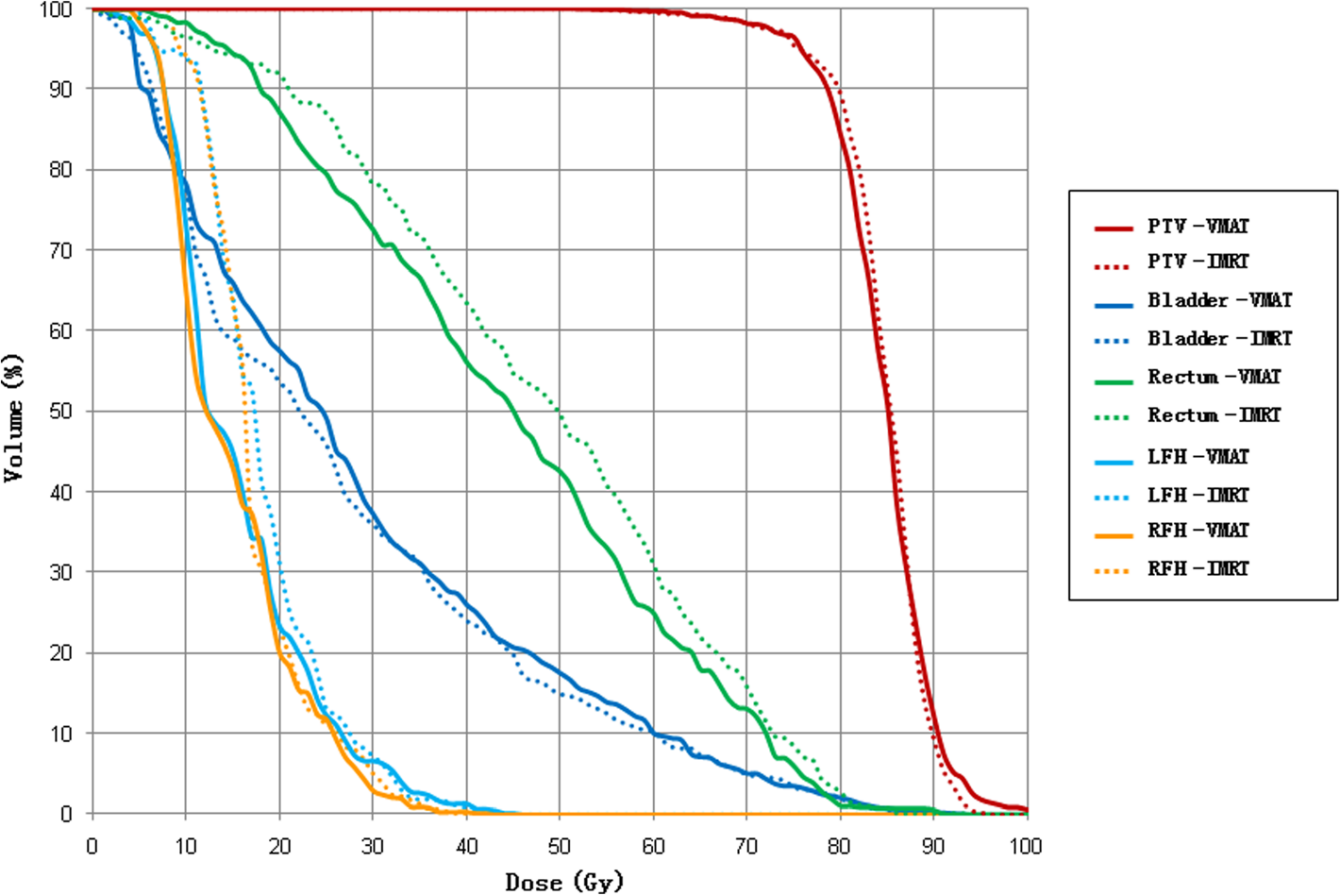
The DVH of prostate case in IMRT plan and VMAT plan

For the prostate case, the objective is to maintain uniform dose (76 Gy) to the PTV while keep the rectal dose at V50 Gy < 50% and maximize sparing of bladder (V50 Gy < 50%) and femoral head (V50 Gy < 5%). Dose distributions for IMRT and VMAT plans are shown in Fig. 6. DVHs for IMRT and VMAT plans are compared as shown in Fig. 7. The VMAT plan is comparable to the corresponding IMRT plan. The coverage of PTV is similar between VMAT and IMRT plans. For VMAT plan, OAR sparing is better for rectum, femoral heads (left and right) while similar for bladder. Dose distribution of VMAT plan is more uniform and has less high dose region in normal tissue. The QS, WE, CI, and HI for both plans are shown in Table 1. The WE is reduced by 12% for VMAT plan, while QS, CI, and HI are similar for both plans. The total number of segments and monitor units for both plans are shown in Table 1. The number of segment is similar for both plans. The total MU for VMAT plan is reduced by 16%. The computation time is 36 s for IMRT plan and 294 s for VMAT plan as shown in Table 1. The estimated delivery time is between 118 s and 236 s based on a Varian Trilogy machine with variable dose rate (300–600 MUs per minute). VMAT_P plan show better plan quality and fewer MUs than VMAT plan, but the number of segments and optimization time are higher.

**Fig. 8:**
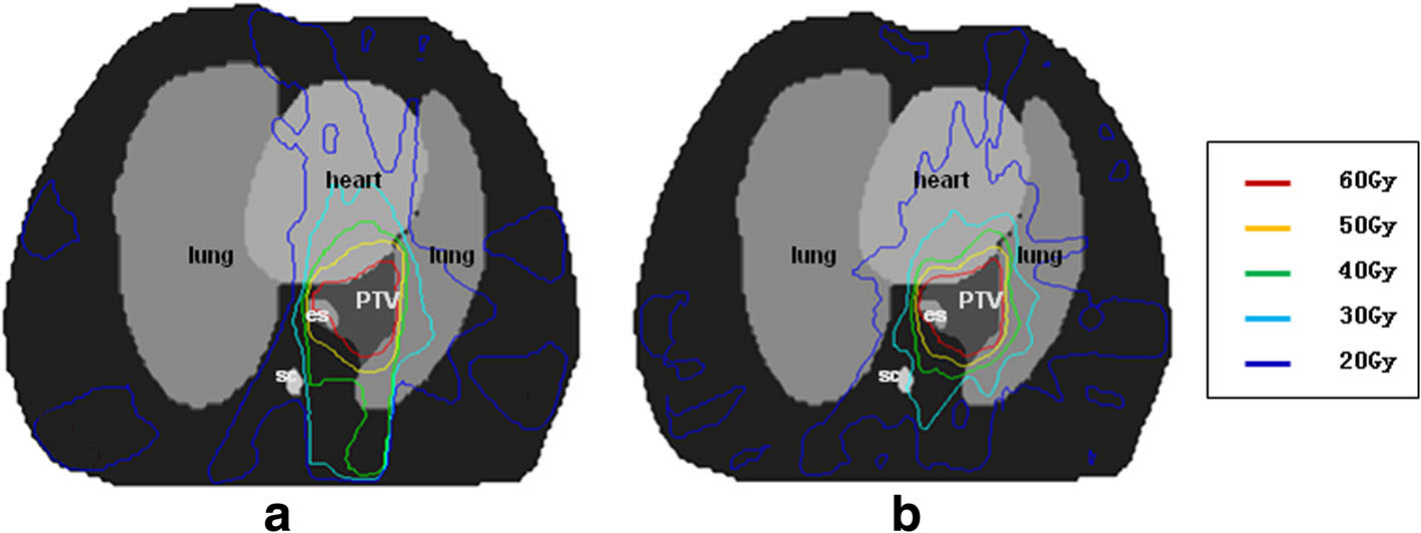
The transaxial plan dose distribution of lung case in (**a**) IMRT plan and (**b**) VMAT plan

**Fig. 9:**
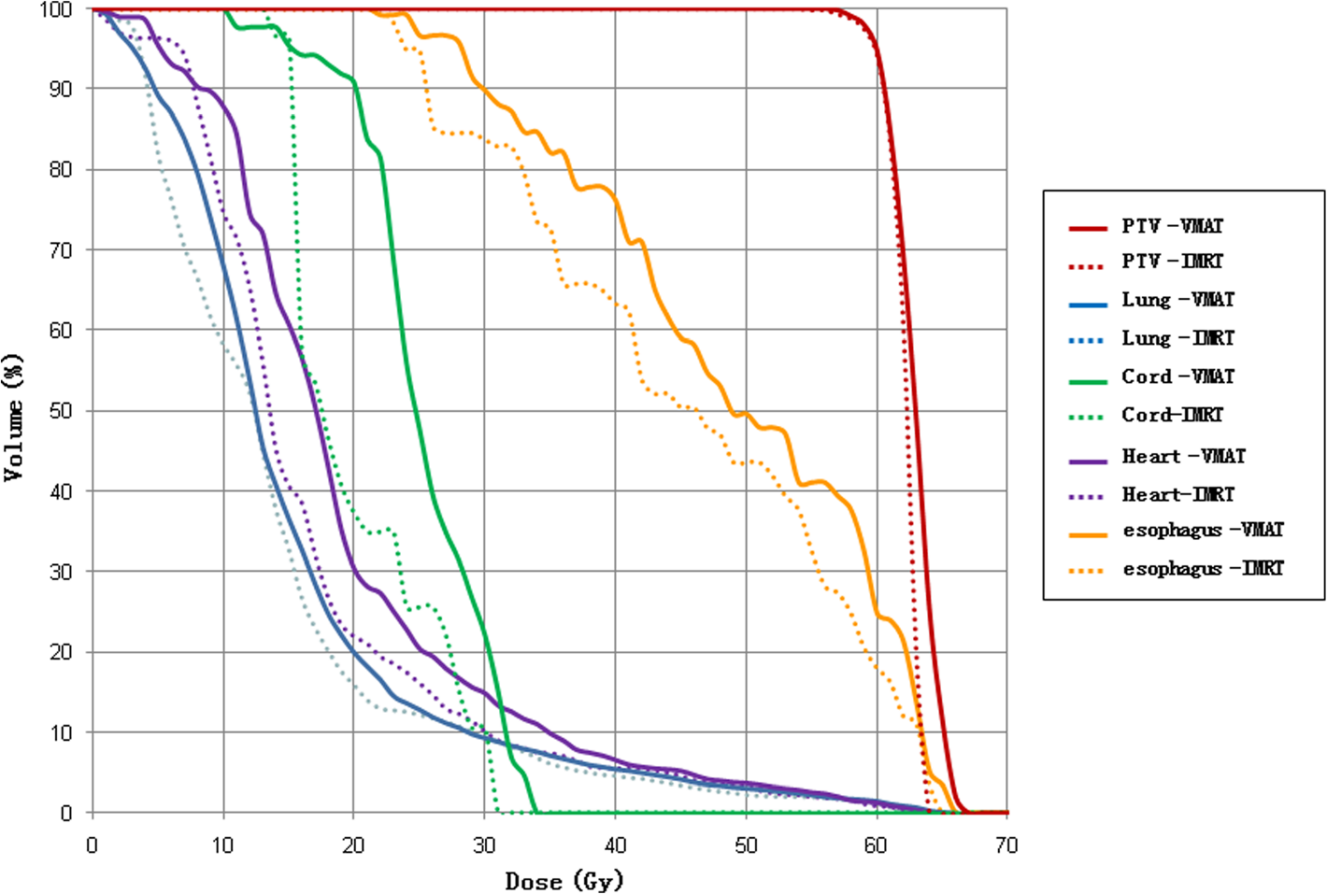
The DVH of lung case in IMRT plan and VMAT plan

For the lung case, the objective is to maintain uniform dose (60 Gy) to the PTV while keep the lung dose at V20 Gy < 25% and maximize the sparing of cord (Dmax< 45 Gy), heart (V30Gy < 40%, V40Gy < 30%) and esophagus (V50Gy < 50%). Dose distributions for IMRT and VMAT plans are shown in Fig. 8. DVHs for IMRT and VMAT plans are compared as shown in Fig. 9. The dose coverage of PTV is similar in both plans. For VMAT plan, dose to heart, esophagus, and cord are slightly higher than those of IMRT plan, while dose to lung is similar. The Dose distribution of the VMAT plan is more uniform and focused on PTV, however the dose distribution of IMRT plan is spread along anterior-posterior direction. The QS, WE, CI, and HI for both plans are shown in Table 1. The WE, QS, CI, and HI are similar for both plans. The total number of segments and monitor units for both plans are shown in Table 1. The number of segment for VMAT plan is reduced by 16%. The total MU is similar for both plans. The computation time is 32 s for IMRT plan and 216 s for VMAT plan as shown in Table 1. The estimated VMAT delivery times are between 116 s and 232 s based on a general linear accelerator with variable dose rate (300–600 MUs per minute). VMAT_P plan show better plan quality and few MUs than VMAT plan, but the optimization time is higher.

## Discussions

The results of clinical case study show that plan quality of VMAT plans is similar to or better than that of traditional IMRT plans. The VMAT plans, in many cases, are able to achieve better OAR sparing compared to IMRT plans. VMAT plan has the potential to tailor high volume low dose regions because of its flexibility to spread out the dose at many beam angles. This character reduces the need of specific structures defined to remove hot spots, thus reduces overall planning effort. Plans consisting of more than 2 arcs are not reported in this work, but it is a viable option in clinical practice. We also tested optimizing all beams in multiple arcs which has slightly improved dosimetric quality. These data are not included in this paper. The dose quality of clinically approved plans made on pinnacle planning system is better. This may be caused by the differences of optimization algorithm, dose calculation engine, dose-volume constraints between pinnacle system and in-house developed system. However, the optimization and delivery time of VMAT plans achieved by our approach is less. It is promising to implement this approach in commercial planning system to accelerate the current process of VMAT optimization.

The selection of beam orientations in initial stages of optimization would affect the final dose considerably. However it could be minimized with increasing number of beams. It was reported that optimizing beam orientations is most valuable for a small numbers of beams (≤5) and the gain diminishes rapidly for higher numbers of beams (≥15) [33]. In several pioneering studies of VMAT optimization it showed 10–15 beams with equal-space would be a better choice in initial stage of optimization [9, 10, 17]. We also tested our algorithm with the different initial beam orientations in the initial stage. It was found that for a higher number of beams (≥15) in the initial stage the final value of objective function is less affected. In the proposed algorithm the number of beams in the initial stage is set to 15, while this value is 10 in RapidArc of Eclipse (Varian Medical System, Palo Alto, CA, USA) and 15 in SmartArc of Pinnacle (Philips Healthcare, Inc., Thornton, CO, USA).

Plan quality can be further improved by increasing the number of beams. However, as more beams included, the computation time for plan optimization and dose calculation could be increased substantially. We speculate that only in certain cases, e.g., with complex target shape requiring substantial leaf motion, it would be beneficial to increase gantry position sampling. It is observed that the number of segments for VMAT plan is less than or similar to that of IMRT plan in three cases. The total MU for VMAT plan is also less than or similar to that of IMRT plan in three cases. Overall, the delivery time for VMAT plan is significantly reduced compared to IMRT plan. Plan optimization times vary case by case but all within 5 min. Compared with the selected commercial treatment planning systems, the running time of plan optimization is reduced by 3–4 times. Also, due to the nature of gradient-based optimization algorithm, the optimization solution is repeatable as long as the initial setting of plan parameters is the same.

The current objective function is quadratic function which can be solved efficiently by gradient decent method. However, for real clinical use it is desired to incorporate more complex objectives and constraints such as dose-volume histogram (DVH) and general equivalent uniform dose (gEUD). For DVH constraints, the current algorithm is applicable with minor modification. Those voxels whose doses exceed the given threshold will be identified when comparing actual DVH to expected DVH. The weights for those voxels will be adjusted automatically in order to minimize the DVH difference. For gEUD constraints, the form of objective function will be changed as described by Wu [34] and can be solved by gradient descent method which is similar to FMD algorithm employed in this study. The workflow shown in Fig. 2 is also feasible for gEUD-based optimization algorithm. It is also possible to use gEUD-based objective to constrain mean dose for normal tissue by setting parameter α to small and positive value. For more challenging clinical cases with complex geometry and spatial distribution of anatomical structures, the overlapped region between PTV and OARs should be handled respectively and supplementary structures would be used.

For the first time the new VMAT plan optimization approach was implemented on an in-house developed planning platform. The progressive sampling strategy is combined with gradient-descent-based FMO algorithm for a high-performance VMAT planning system. This work demonstrates a new way to transform the existing optimization algorithms originally designed for IMRT to the new algorithm for VMAT planning. Currently, the dynamic arcs are planned with a single 360° with 2° angle spacing, varying dose rate, and constant gantry speed. Sensitivity to parameters such collimator angle, couch angle, arc length, and delivery time are not explored methodically. However these parameters may provide plan quality improvements for some cases. In addition, there may be a need for multiple arcs for cases not included in this study. Determining optimal use of these parameters and the potential automation of the parameter settings are subjects to ongoing investigation. It is also necessary to perform phantom verification in order to implement this algorithm for clinical application.

## Conclusion

A new approach was developed which is based on fast gradient descent algorithm and mixed integer programming technique to provide a high-performance VMAT planning. Results from clinical case studies demonstrated that plan quality of VMAT plans is similar to or better than that of IMRT plans. The optimization time and the number of segments are reduced considerably. This work demonstrates a way to transform the existing optimization algorithms originally designed for IMRT to the new algorithm designed for VMAT planning.

## Appendix 1

The objective function of classic FMD is defined as below.

1 $$ \min \kern0.5em f\left(\mathbf{x}\right)=\sum \limits_i\sum \limits_j\sum \limits_k{W}_{ijk}{\left({D}_{ijk}-{P}_{ijk}\right)}^2 $$

$$ \mathrm{where}\kern0.5em {D}_{ijk}=\sum \limits_{n=1}^N{A}_{n, ijk}{x}_n $$

*N* is the total number of beamlet and *x*_*n*_ is the fluence of beamlet *n*. *D*_*ijk*_ denotes the dose absorbed by voxel *v*_*ijk*_. *A*_*n,ijk*_ is the dose deposition coefficient defining the dose contribution per unit fluence of beamlet *n* to voxel (*i*, *j*, *k*). *P*_*ijk*_ is the constraint doses for target volume (TV), critical organs (CO), and normal tissue (NT). *W*_*ijk*_ is the weighting factors assigned for TV, CO, and NT. *P*_*ijk*_ and *W*_*ijk*_ are predefined according to clinical requirement. The objective is to find minimum of *f*(***x***) subject to *x*_*n*_ > 0 for all beamlet *n∈N*. A non-synchronous updating scheme as shown below is used which allows only one beamlet adjusted at a time.

2 $$ {x}_m\left(l+1\right)={x}_m(l)+\Delta {x}_m,m\in N $$

Here

$$ \Delta {x}_m=\sum \limits_{n=1}^N{B}_{mn}{x}_n(l)+{C}_m $$, $$ {B}_{mn}=\frac{-\sum \limits_i\sum \limits_j\sum \limits_k{W}_{ijk}{A}_{m, ijk}{A}_{n, ijk,}}{\sum \limits_i\sum \limits_j\sum \limits_k{W}_{ijk}{A}_{m, ijk}^2} $$$$ {C}_m=\frac{\sum \limits_i\sum \limits_j\sum \limits_k{W}_{ijk}{A}_{m, ijk}{P}_{ijk}}{\sum \limits_i\sum \limits_j\sum \limits_k{W}_{ijk}{A}_{m, ijk}^2} $$.

*l* is the iteration number and *m* is the index of beamlet adjusted in *l*–th iteration. Only one beamlet adjusted at one iteration. If all beamlets are adjusted one time, there are *N* iterations and it is called one cycle. Usually it takes few cycles (~ 10) to reach a satisfactory solution for one beam set.

To accommodate the progressive sampling strategy of VMAT optimization, FMD algorithm was modified to account for the increasing number of beams. The optimization process starts from the initial beam set consisting of few beams (usually 5), and then the fluence maps are achieved by FMO and processed by leaf sequencing. The beam set keeps expanding and new beams are added. The beams from previous beam set are called old beams and the beams newly added are called new beams. In a given beam set, the fluence maps of old beams are fixed and the fluence maps of new beams are optimized by FMO for several cycles. The beam set will continuously grow until the maximal number of beams reaches. For each beam set, there are many beamlets corresponding to the given number of beams. Assuming there are *N*^*T*^ beamlets in *T*-th beam set and *N*^*T*^ = *N*^*T*^_*new*_ + *N*^*T*^_*old*_ where *N*^*T*^_*new*_ and *N*^*T*^_*old*_ are the numbers of beamlets for new and old beams. *x*_*r*_^*new*^ denotes the fluence of *r*-th beamlet of new beam while *x*_*s*_^*old*^ denotes the fluence of *s*-th beamlet of old beam. For a given voxel *v*_*ijk*_, its dose is calculated based on the fluences of all *x*_*r*_^*new*^ and *x*_*s*_^*old*^.

3 $$ {D}_{ijk}=\sum \limits_{r=1}^{N_{new}^T}{A}_{r, ijk}{x}_r^{new}+\sum \limits_{s=1}^{N_{old}^T}{A}_{s, ijk}{x}_s^{old} $$

Here *r* and *s* are the index of beamlets of new and old beams. Accordingly, the objective function (1) can be rewritten as below.

4 $$ f(x)=\sum \limits_i\sum \limits_j\sum \limits_k{w}_{ijk}{\left[{P}_{ijk}-\left(\sum \limits_{r=1}^{N_{new}^T}{A}_{r, ijk}{x}_r^{new}+\sum \limits_{s=1}^{N_{old}^T}{A}_{s, ijk}{x}_s^{old}\right)\right]}^2 $$

Alternatively, it can be expressed as.

5 $$ f(x)=\sum \limits_i\sum \limits_j\sum \limits_k{w}_{ijk}{\left[\left({P}_{ijk}-\sum \limits_{r=1}^{N_{new}^T}{A}_{r, ijk}{x}_r^{new}\right)-\sum \limits_{s=1}^{N_{old}^T}{A}_{s, ijk}{x}_s^{old}\right]}^2 $$

Then the expression (5) can be extended as.

6 $$ f(x)=\sum \limits_i\sum \limits_j\sum \limits_k\left[{w}_{ijk}{\left({P}_{ijk}-\sum \limits_{r=1}^{N_{new}^T}{A}_{r, ijk}{x}_r^{new}\right)}^2-2{w}_{ijk}\left({P}_{ijk}-\sum \limits_{r=1}^{N_{new}^T}{A}_{r, ijk}{x}_r^{new}\right)\sum \limits_{s=1}^{N_{old}^T}{A}_{s, ijk}{x}_s^{old}+{w}_{ijk}{\left(\sum \limits_{s=1}^{N_{old}^T}{A}_{s, ijk}{x}_s^{old}\right)}^2\right] $$

7 $$ {\displaystyle \begin{array}{l}f(x)=\sum \limits_i\sum \limits_j\sum \limits_k{w}_{ijk}{\left({P}_{ijk}-\sum \limits_{r=1}^{N_{new}^T}{A}_{r, ijk}{x}_r^{new}\right)}^2-\sum \limits_i\sum \limits_j\sum \limits_k2{w}_{ijk}\left[\left({P}_{ijk}-\sum \limits_{r=1}^{N_{new}^T}{A}_{r, ijk}{x}_r^{new}\right)\sum \limits_{s=1}^{N_{old}^T}{A}_{s, ijk}{x}_s^{old}\right]\\ {}+\sum \limits_i\sum \limits_j\sum \limits_k{w}_{ijk}{\left(\sum \limits_{s=1}^{N_{old}^T}{A}_{s, ijk}{x}_s^{old}\right)}^2\end{array}} $$

To minimize the objective function in (2) with respect to the fluence of *m*-th beamlet, its gradient at *l + 1* iteration should be zero.

8 $$ \frac{\partial f\left[x\left(l+1\right)\right]}{\partial {x}_m}=0 $$

The gradient of objective function at *l + 1* iteration is expressed as.

9 $$ \frac{\partial f\left[x\left(l+1\right)\right]}{\partial {x}_m}=\sum \limits_i\sum \limits_j\sum \limits_k2{w}_{ijk}\left({P}_{ijk}-\sum \limits_{r=1}^{N_{new}^T}{A}_{r, ijk}{x}_r^{new}\left(l+1\right)\right)\left(-{A}_{m, ijk}\right)-\sum \limits_i\sum \limits_j\sum \limits_k\left[2{w}_{ijk}\sum \limits_{s=1}^{N_{old}^T}{A}_{s, ijk}{x}_s^{old}\left(-{A}_{m, ijk}\right)\right] $$

For m∈, the update formula (2) is.

10 $$ {x}_m^{new}\left(l+1\right)={x}_m^{new}(l)+\Delta {x}_m^{new},m\in {N}_T^{new} $$

Put (8) and (9) together we have

11 $$ \sum \limits_i\sum \limits_j\sum \limits_k2{w}_{ijk}\left({P}_{ijk}-\sum \limits_{r=1}^{N_{new}^T}{A}_{r, ijk}{x}_r^{new}\left(l+1\right)\right)\left(-{A}_{m, ijk}\right)-\sum \limits_i\sum \limits_j\sum \limits_k\left[2{w}_{ijk}\sum \limits_{s=1}^{N_{old}^T}{A}_{s, ijk}{x}_s^{old}\left(-{A}_{m, ijk}\right)\right]=0. $$

Replace ${x}_r^{new}\left(l+1\right)$ in (11) with [${x}_r^{new}(l)$+Δ*x*] in (10), we have

12 $$ \sum \limits_i\sum \limits_j\sum \limits_k{w}_{ijk}{A}_{m, ijk}\left\{{P}_{ijk}-\left[\sum \limits_{r=1}^{N_{new}^T}{A}_{r, ijk}{x}_r^{new}(l)+{A}_{m, ijk}\Delta {x}_m\right]\right\}=\sum \limits_i\sum \limits_j\sum \limits_k{w}_{ijk}{A}_{m, ijk}\sum \limits_{s=1}^{N_{old}^T}{A}_{s, ijk}{x}_s^{old} $$

Equation (12) can be expressed as

13 $$ {\displaystyle \begin{array}{l}\sum \limits_i\sum \limits_j\sum \limits_k{w}_{ijk}{A}_{m, ijk}\left\{{P}_{ijk}\right\}-\sum \limits_i\sum \limits_j\sum \limits_k{w}_{ijk}{A}_{m, ijk}\sum \limits_{r=1}^{N_{new}^T}\left[{A}_{r, ijk}{x}_r^{new}(l)\right]-\sum \limits_i\sum \limits_j\sum \limits_k{w}_{ijk}{A}_{m, ijk}{A}_{m, ijk}\Delta {x}_m\\ {}=\sum \limits_i\sum \limits_j\sum \limits_k{w}_{ijk}{A}_{m, ijk}\sum \limits_{s=1}^{N_{old}^T}{A}_{s, ijk}{x}_s^{old}.\end{array}} $$

With proper arrangement of both sides of Eq. (13), it is

14 $$ {\displaystyle \begin{array}{l}\sum \limits_i\sum \limits_j\sum \limits_k{w}_{ijk}{P}_{ijk}{A}_{m, ijk}-\sum \limits_i\sum \limits_j\sum \limits_k{w}_{ijk}\sum \limits_{r=1}^{N_{new}^T}{A}_{m, ijk}{A}_{r, ijk}{x}_r^{new}(l)-\sum \limits_i\sum \limits_j\sum \limits_k{w}_{ijk}\sum \limits_{s=1}^{N_{old}^T}{A}_{m, ijk}{A}_{s, ijk}{x}_s^{old}\\ {}=\sum \limits_i\sum \limits_j\sum \limits_k{w}_{ijk}{A}_{m, ijk}^2\Delta {x}_m.\end{array}} $$

Then Δ*x*_*m*_is calculated as

15 $$ \Delta {x}_m=\frac{\sum \limits_i\sum \limits_j\sum \limits_k{w}_{ijk}{P}_{ijk}{A}_{m, ijk}-\sum \limits_i\sum \limits_j\sum \limits_k{w}_{ijk}\sum \limits_{r=1}^{N_{new}^T}{A}_{m, ijk}{A}_{r, ijk}{x}_r^{new}(l)-\sum \limits_i\sum \limits_j\sum \limits_k{w}_{ijk}\sum \limits_{s=1}^{N_{old}^T}{A}_{m, ijk}{A}_{s, ijk}{x}_s^{old}}{\sum \limits_i\sum \limits_j\sum \limits_k{w}_{ijk}{A}_{m, ijk}^2}. $$

15a $$ {B}_m^{new}=-\frac{\sum \limits_i\sum \limits_j\sum \limits_k{w}_{ijk}\sum \limits_{n_1=1}^{N_{new}^T}{A}_{r, ijk}{x}_r(l){A}_{m, ijk}}{\sum \limits_i\sum \limits_j\sum \limits_k{w}_{ijk}{A}_{m, ijk}^2}=-\sum \limits_{r=1}^{N_{new}^T}{x}_r^{new}(l)\frac{\sum \limits_i\sum \limits_j\sum \limits_k{w}_{ijk}{A}_{m, ijk}{A}_{r, ijk}}{\sum \limits_i\sum \limits_j\sum \limits_k{w}_{ijk}{A}_{m, ijk}^2}. $$

(15a) can also be expressed as ${B}_m^{new}=\sum \limits_{r=1}^{N_{new}^T}{x}_r^{new}(l){B}_{mr}^{new}$,where ${B}_{mr}^{new}=-\frac{\sum \limits_i\sum \limits_j\sum \limits_k{w}_{ijk}{A}_{m, ijk}{A}_{r, ijk}}{\sum \limits_i\sum \limits_j\sum \limits_k{w}_{ijk}{A}_{m, ijk}^2},r\in {N}_{new}^T$.

15b $$ {B}_m^{old}=\frac{-\sum \limits_i\sum \limits_j\sum \limits_k{w}_{ijk}\sum \limits_{s=1}^{N_{old}^T}{A}_{s, ijk}{x}_s^{old}{A}_{m, ijk}}{\sum \limits_i\sum \limits_j\sum \limits_k{w}_{ijk}{A}_{m, ijk}^2}=\sum \limits_{s=1}^{N_{old}^T}{x}_s^{old}\frac{-\sum \limits_i\sum \limits_j\sum \limits_k{w}_{ijk}{A}_{m, ijk}{A}_{s, ijk}}{\sum \limits_i\sum \limits_j\sum \limits_k{w}_{ijk}{A}_{m, ijk}^2} $$

(15b) can also be expressed as ${B}_m^{old}=\sum \limits_{s=1}^{N_{old}^T}{x}_s^{old}{B}_{ms}^{old}$, where ${B}_{ms}^{old}=\frac{-\sum \limits_i\sum \limits_j\sum \limits_k{w}_{ijk}{A}_{m, ijk}{A}_{s, ijk}}{\sum \limits_i\sum \limits_j\sum \limits_k{w}_{ijk}{A}_{m, ijk}^2},s\in {N}_{old}^T$.

15c $$ {C}_m=\frac{\sum \limits_i\sum \limits_j\sum \limits_k{w}_{ijk}{A}_{m, ijk}{P}_{ijk}}{\sum \limits_i\sum \limits_j\sum \limits_k{w}_{ijk}{A}_{m, ijk}^2} $$

Finally, the updating formula is below.

16 $$ {x}_m\left(l+1\right)={x}_m(l)+\sum \limits_{r=1}^{N_{new}^T}{B}_{mr}^{new}{x}_r^{new}(l)+\sum \limits_{s=1}^{N_{old}^T}{B}_{ms}^{old}{x}_s^{old}+{C}_m,\kern0.5em m\in {N}_{new}^T $$

Note that ${x}_s^{old}$ is solved in *1*-th to *T*-1-th beam set and constant in *T*-th beam set, while ${x}_r^{new}$ is variable to be solved in *T*-th beam set. *C*_*m*_is calculated based on *w*_*ijk*_,*A*_*m*, *ijk*_and *P*_*ijk*_ which can be pre-computed prior to the optimization of *1*-th beam set. ${B}_m^{old}$ is calculated based on constants*w*_*ijk*_,*A*_*m*, *ijk*_,*P*_*ijk*_ and ${x}_s^{old}$. ${B}_m^{old}$ can be pre-computed prior to the optimization of *T*-th beam set. ${B}_m^{new}$ is calculated based on constants*w*_*ijk*_,*A*_*m*, *ijk*_,*P*_*ijk*_ and variable ${x}_r^{new}$. ${B}_m^{old}$ can’t be pre-computed and should be calculated online.

## Appendix 2

The objective function of the MILP model is defined as below.

1 $$ \min \kern0.5em \sum \limits_{i=1}^N\sum \limits_{j=1}^M\left({I}_{ij}^c-{A}_{ij}^c\right) $$

Here ${I}_{ij}^c$ is the original fluence of bixel (beam pixel) after FMO, and ${A}_{ij}^c$is the unknown fluence of bixel for the single aperture. *N* and *M* are the dimensions of fluence map indexed with *ij*. For achieving the best uniform fluence map*A*^*c*^in approximating non-uniform fluence map*I*^*c*^, the following constraints are required.

2 $$ {l}_{ij}^c+{r}_{ij}^c=1-{b}_{ij}^c $$

3 $$ {l}_{ij+1}^c\le {l}_{ij}^c $$

4 $$ {r}_{ij+1}^c\ge {r}_{ij}^c $$

5 $$ {A}_{ij}^c={A}^c{b}_{ij}^c $$

6 $$ {A}_{ij}^c\le {I}_{ij}^c $$

7 $$ \left|\sum \limits_{j=1}^M{l}_{ij}^c-\sum \limits_{j=1}^M{l}_{ij}^p\right|\le \frac{v_{\mathrm{max}}}{s^{\phi }}\Delta \phi $$

8 $$ \left|\sum \limits_{j=1}^M{l}_{ij}^c-\sum \limits_{j=1}^M{l}_{ij}^f\right|\le \frac{v_{\mathrm{max}}}{s^{\phi }}\Delta \phi $$

9 $$ \left|\sum \limits_{j=1}^M{r}_{ij}^c-\sum \limits_{j=1}^M{r}_{ij}^p\right|\le \frac{v_{\mathrm{max}}}{s^{\phi }}\Delta \phi $$

10 $$ \left|\sum \limits_{j=1}^M{r}_{ij}^c-\sum \limits_{j=1}^M{r}_{ij}^f\right|\le \frac{v_{\mathrm{max}}}{s^{\phi }}\Delta \phi $$

The superscripts *c*, *p*, and *f* represent current beam, previous beam and following beam.

The constants in the above constraints are defined as:

*d*_max_: Maximal dose rate.

*v*_max_: Maximal leaf speed.

*s*^*ϕ*^: Gantry angular speed.

Δ*ϕ*: Angular spacing between two beams.

${I}_{ij}^c$: Contains the original fluence of bixel *ij* resulted by FMO.

*A*^*c*^: Maximal fluence of current beam and is less than $\frac{v_{\mathrm{max}}}{s^{\phi }}{d}_{\mathrm{max}}$.

The variables in the above constraints are defined as:

${l}_{ij}^c$: Binary variable indicates whether bixel *ij* of current beam is blocked by left leaf or not (1: close; 0: open).

${r}_{ij}^c$: Binary variable indicates whether bixel *ij* of current beam is blocked by right leaf or not (1: close; 0: open).

${b}_{ij}^c$: Binary variable indicates whether bixel *ij* of current beam is open or not (1: open; 0: close).

${A}_{ij}^c$: Float variable contains the fluence of bixel *ij*.

${l}_{ij}^p$: Binary variable indicates whether bixel *ij* of pervious beam is blocked by left leaf or not (1: close; 0: open).

${l}_{ij}^f$: Binary variable indicates whether bixel *ij* of following beam is blocked by left leaf or not (1: close; 0: open).

${r}_{ij}^p$: Binary variable indicates whether bixel *ij* of pervious beam is blocked by right leaf or not (1: close; 0: open).

${r}_{ij}^f$: Binary variable indicates whether bixel *ij* of following beam is blocked by right leaf or not (1: close; 0: open).

The meanings of constraints are explained as below.

Constraint (2): requires bixel *ij* either blocked by leaf or open.

Constraint (3): requires mechanical continuity of left leaf.

Constraint (4): requires mechanical continuity of right leaf.

Constraint (5): requires fluence of beam either zero or fluence *A*^*c*^.

Constraint (6): requires fluence of beam is less than fluence *I*_*ij*_^*c*^.

Constraint (7): requires the distance difference between left leaf positions in current and previous beams less than $\frac{v_{\mathrm{max}}}{s^{\phi }}\Delta \phi$, which is the maximal distance that a leaf can travel between two neighboring control points or angles.

Constraint (8): requires the distance difference between left leaf positions in current and following beams less than $\frac{v_{\mathrm{max}}}{s^{\phi }}\Delta \phi$ which is the maximal distance that a leaf can travel between two neighboring control points or angles.

Constraint (9): requires the distance difference between right leaf positions in current and previous beams less than $\frac{v_{\mathrm{max}}}{s^{\phi }}\Delta \phi$, which is the maximal distance that a leaf can travel between two neighboring control points or angles.

Constraint (10): requires the distance difference between right leaf positions in current and following beams less than $\frac{v_{\mathrm{max}}}{s^{\phi }}\Delta \phi$, which is the maximal distance that a leaf can travel between two neighboring control points or angles.

## Abbreviations

CI: Conformity index
CO: Critical organ
DAO: Direct aperture optimization
DVH: Dose volume histogram
DVH: Dose-volume histogram
FMD: Fast monotonic descent
FMO: Fluence map optimization
gEUD: general equivalent uniform dose
HI: Homogeneity index
IMAT: Intensity modulated arc therapy
IMRT: Intensity modulated radiation therapy
MILP: Mixed integer linear programming
MLC: Multiple leaf collimator
MU: Monitor unit
NT: Normal tissue
OAR: Organ at risk
PTV: Planning target volume
QS: Quality score
ROI: Region of interesting
TV: Target volume
VMAT: Volumetric modulated arc therapy
WE: Weighted error

## References

[1] Teoh M, Clark CH, Wood K, Whitaker S, Nisbet A (2011). Volumetric modulated arc therapy: a review of current literature and clinical use in practice. Br J Radiol.

[2] Bedford JL (2009). Treatment planning for volumetric modulated arc therapy. Med Phys.

[3] Rao M, Yang W, Chen F, Sheng K, Ye J, Mehta V, Shepard D, Cao D (2010). Comparison of Elekta VMAT with helical tomotherapy and fixed field IMRT: plan quality, delivery efficiency and accuracy. Med Phys.

[4] Yu CX, Tang G (2011). Intensity-modulated arc therapy: principles, technologies and clinical implementation. Phys Med Biol.

[5] Yu CX (1995). Intensity-modulated arc therapy with dynamic multileaf collimation: an alternative to tomotherapy. Phys Med Biol.

[6] Shepard DM, Earl MA, Li XA, Naqvi S, Yu C (2002). Direct aperture optimization: a turnkey solution for step-and-shoot IMRT. Med Phys.

[7] Crooks SM, Wu X, Takita C, Watzich M, Xing L (2003). Aperture modulated arc therapy. Phys Med Biol.

[8] Bortfeld T, Webb S (2009). Single-arc IMRT?. Phys Med Biol.

[9] Otto K (2008). Volumetric modulated arc therapy: IMRT in a single gantry arc. Med Phys.

[10] Vanetti E, Nicolini G, Nord J, Peltola J, Clivio A, Fogliata A, Cozzi L (2011). On the role of the optimization algorithm of RapidArc(®) volumetric modulated arc therapy on plan quality and efficiency. Med Phys.

[11] Verbakel WF, Cuijpers JP, Hoffmans D, Bieker M, Slotman BJ, Senan S (2009). Volumetric intensity-modulated arc therapy vs. conventional IMRT in head-and-neck cancer: a comparative planning and dosimetric study. Int J Radiat Oncol Biol Phys.

[12] Unkelbach J, Bortfeld T, Craft D, Alber M, Bangert M, Bokrantz R, Chen D, Li R, Xing L, Men C, Nill S, Papp D, Romeijn E, Salari E (2015). Optimization approaches to volumetric modulated arc therapy planning. Med Phys.

[13] Rao M, Cao D, Chen F, Ye J, Mehta V, Wong T, Shepard D (2010). Comparison of anatomy-based, fluence-based and aperture-based treatment planning approaches for VMAT. Phys Med Biol.

[14] Wang C, Luan S, Tang G, Chen DZ, Earl MA, Yu CX (2008). Arc-modulated radiation therapy (AMRT): a single-arc form of intensity-modulated arc therapy. Phys Med Biol.

[15] Ulrich S, Nill S, Oelfke U (2007). Development of an optimization concept for arc-modulated cone beam therapy. Phys Med Biol.

[16] Cameron C (2005). Sweeping-window arc therapy: an implementation of rotational IMRT with automatic beam-weight calculation. Phys Med Biol.

[17] Bzdusek K, Friberger H, Eriksson K, Hardemark B, Robinson D, Kaus M (2009). Development and evaluation of an efficient approach to volumetric arc therapy planning. Med Phys.

[18] Men C, Romeijn HE, Jia X, Jiang SB (2010). Ultrafast treatment plan optimization for volumetric modulated arc therapy (VMAT). Med Phys.

[19] Papp D, Unkelbach J (2014). Direct leaf trajectory optimization for volumetric modulated arc therapy planning with sliding window delivery. Med Phys.

[20] Craft D, McQuaid D, Wala J, Chen W, Salari E, Bortfeld T (2012). Multicriteria VMAT optimization. Med Phys.

[21] Matuszak MM, Steers JM, Long T, McShan DL, Fraass BA, Romeijn HE, Ten Haken RK (2013). FusionArc optimization: a hybrid volumetric modulated arc therapy (VMAT) and intensity modulated radiation therapy (IMRT) planning strategy. Med Phys.

[22] Nguyen D, Lyu Q, Ruan D, O'Connor D, Low DA, Sheng KA (2016). Comprehensive formulation for volumetric modulated arc therapy planning. Med Phys.

[23] Yan H, Yin FF, Guan HQ, Kim JH (2003). AI-guided parameter optimization in inverse treatment planning. Phys Med Biol.

[24] Li RP, Yin FF (2000). Optimization of inverse treatment planning using a fuzzy weight function. Med Phys.

[25] Rowbottom CG1, Webb S, Oldham M. Improvements in prostate radiotherapy from the customization of beam directions. Med Phys 1998; 25(7 Pt 1):1171–1179. 10.1118/1.598308 9682202

[26] Xia P, Hwang AB, Verhey LJ (2002). A leaf sequencing algorithm to enlarge treatment field length in IMRT. Med Phys.

[27] Langer M, Thai V, Papiez L (2001). Improved leaf sequencing reduces segments or monitor units needed to deliver IMRT using multileaf collimators. Med Phys.

[28] Chen Y, Hou Q, Galvin JM (2004). A graph-searching method for MLC leaf sequencing under constraints. Med Phys.

[29] Dai J, Zhu Y (2001). Minimizing the number of segments in a delivery sequence for intensity-modulated radiation therapy with a multileaf collimator. Med Phys.

[30] Yang R, Dai J, Yang Y, Hu Y (2006). Beam orientation optimization for intensity-modulated radiation therapy using mixed integer programming. Phys Med Biol.

[31] Zhu X, Thongphiew D, McMahon R, Li T, Chankong V, Yin FF, Arc-modulated WQJ (2010). Radiation therapy based on linear models. Phys Med Biol.

[32] Bohsung J, Gillis S, Arrans R, Bakai A, De Wagter C, Knöös T, Mijnheer BJ, Paiusco M, Perrin BA, Welleweerd H, Williams P (2005). IMRT treatment planning:- a comparative inter-system and inter-Centre planning exercise of the ESTRO QUASIMODO group. Radiother Oncol.

[33] Stein J, Mohan R, Wang XH, Bortfeld T, Wu Q, Preiser K, Ling CC, Schlegel W (1997). Number and orientations of beams in intensity-modulated radiation treatments. Med Phys.

[34] Wu Q, Mohan R, Niemierko A, Schmidt-Ullrich R (2002). Optimization of intensity-modulated radiotherapy plans based on the equivalent uniform dose. Int J Radiat Oncol Biol Phys.

